# Supplementary figures and images for: Differential scanning fluorimetric analysis of the amino-acid binding to taste receptor using a model receptor protein, the ligand-binding domain of fish T1r2a/T1r3
Source: PLoS One. 2019 Oct 4;14(10):e0218909. doi: 10.1371/journal.pone.0218909 (PMC6777825; doi:10.1371/journal.pone.0218909)

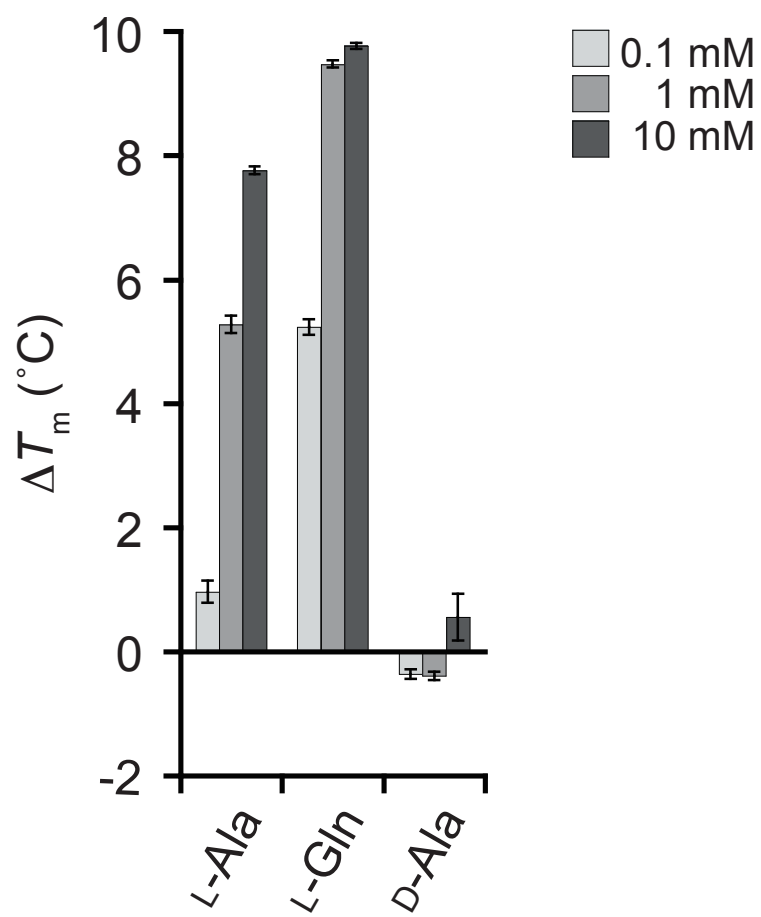

Supplement: S1 Fig — The average ΔTm of T1r2a/T1r3LBD in the presence of 0.1, 1, and 10 mM of l-glutamine, l-alanine, and d-alanine are shown. Error bars, s.e.m. (n = 4). The T0 in this condition was determined as 56.3 ± 0.06 °C (n = 12). Please also see Fig 3A. (PDF) [file pone.0218909.s001.pdf]

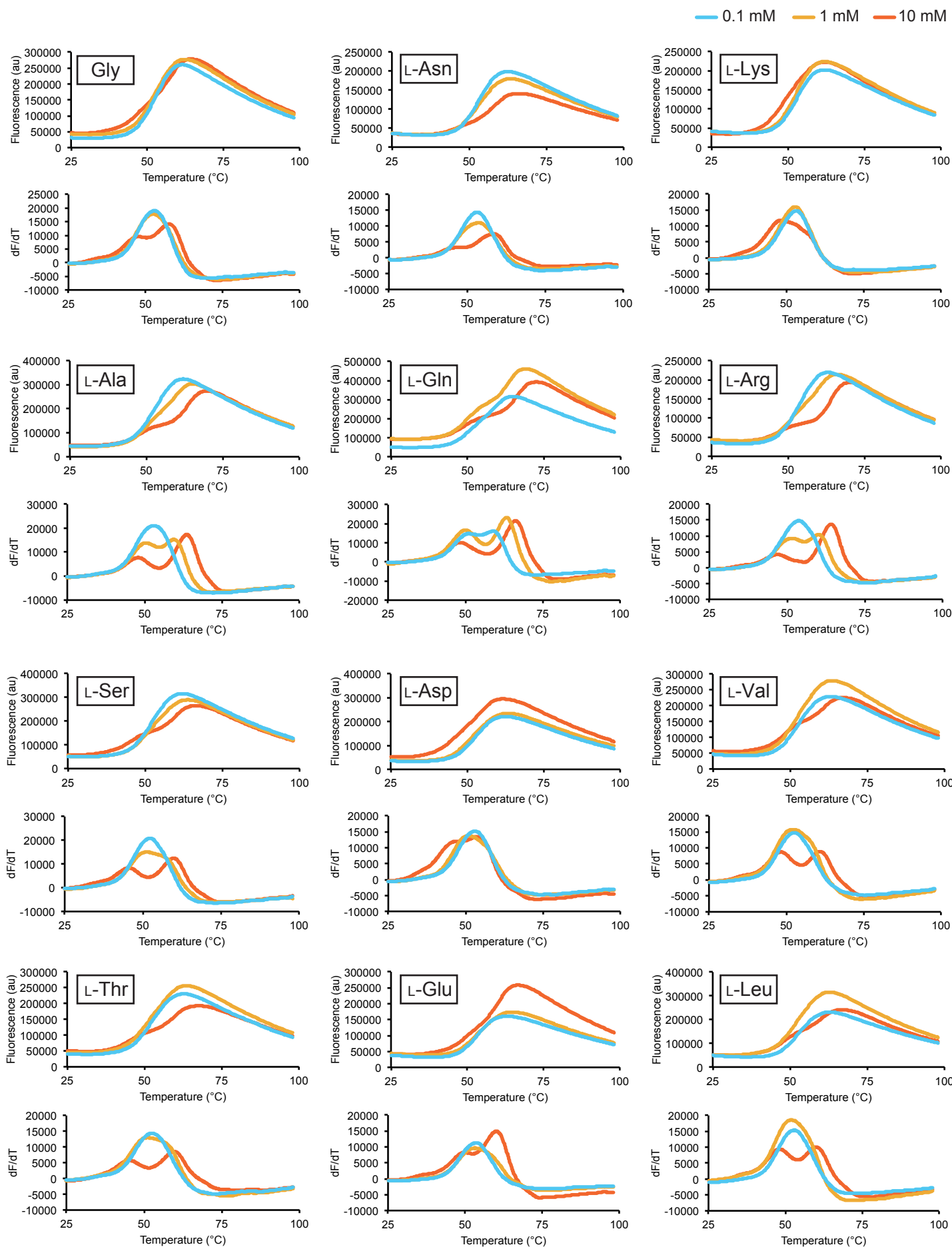

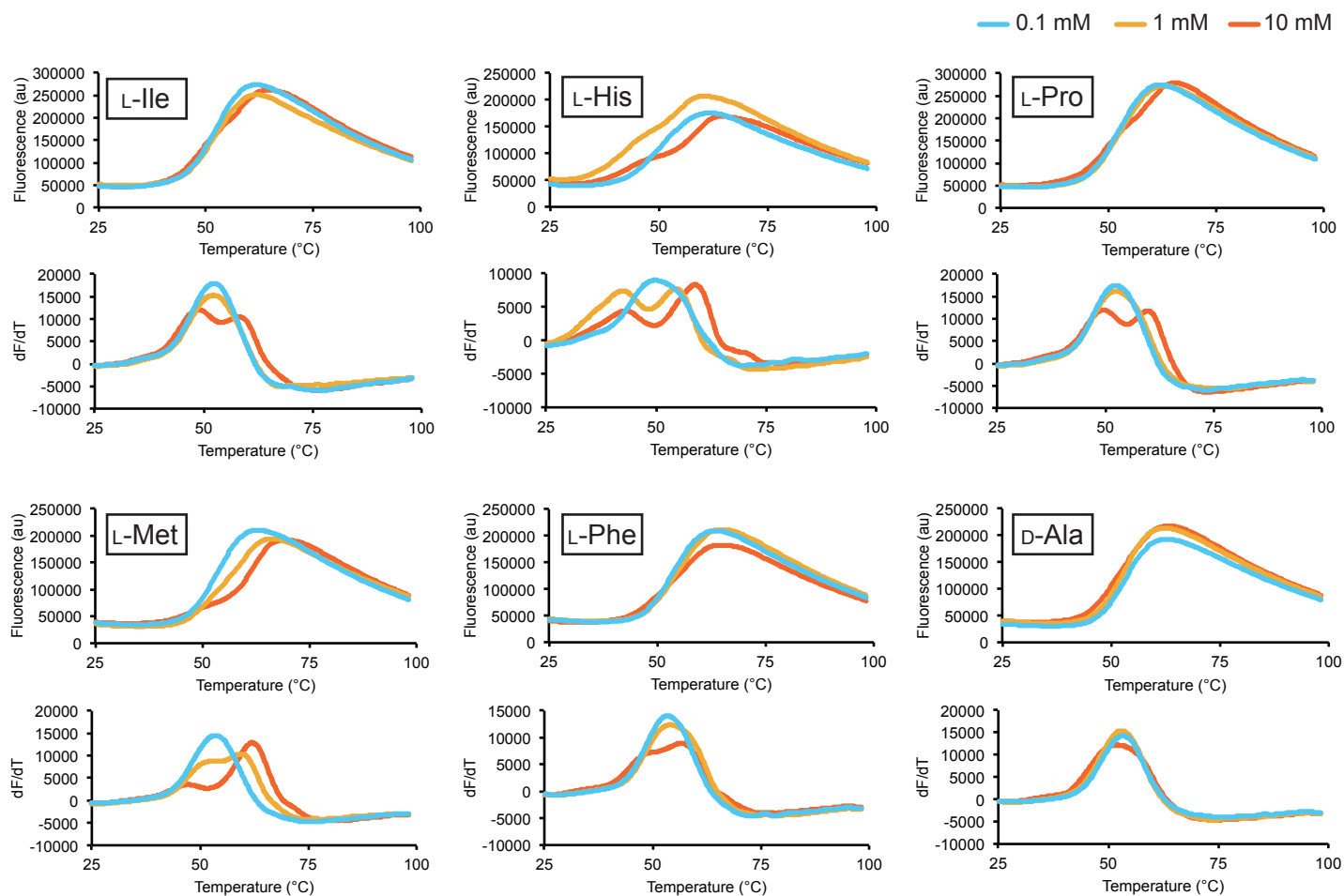

Supplement: S2 Fig — (PDF) [file pone.0218909.s002.pdf]

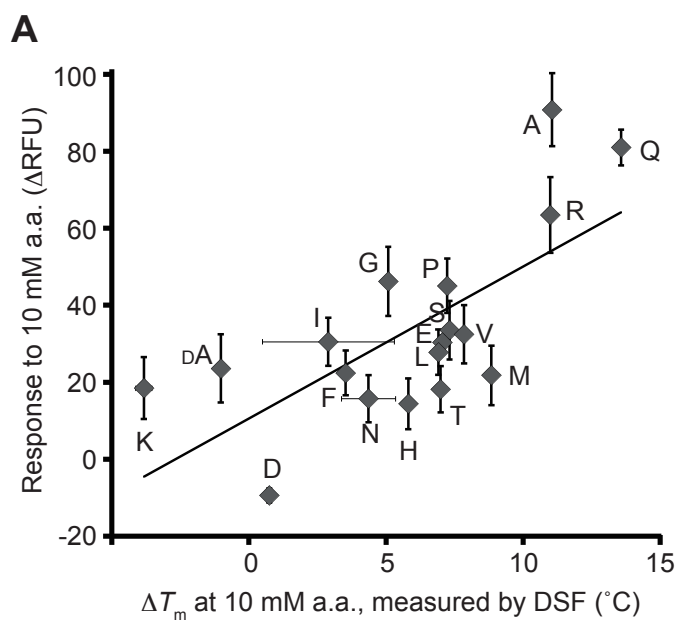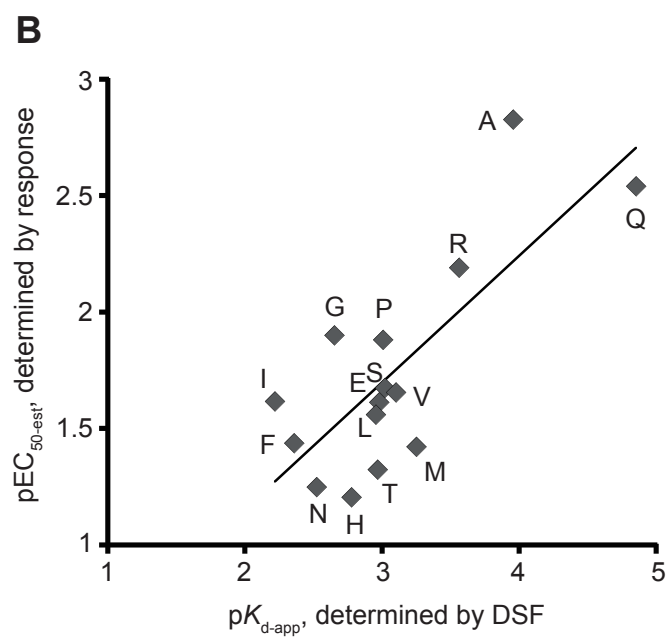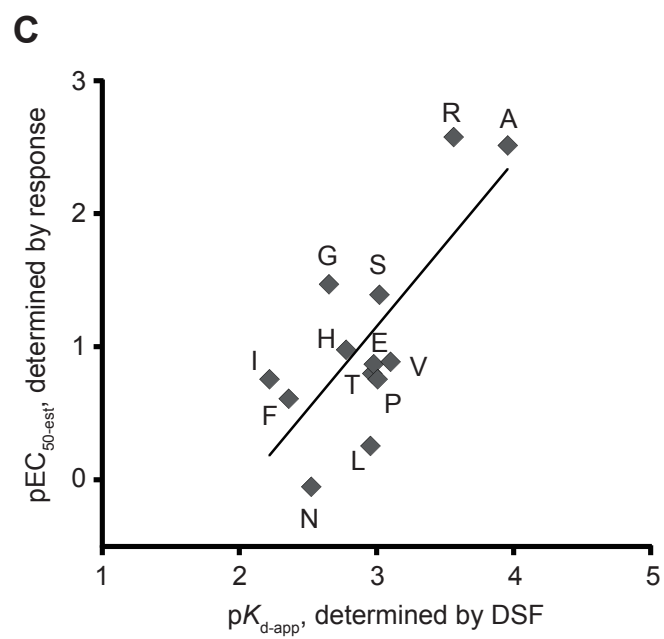

Supplement: S3 Fig — (A) The thermal stabilization of LBD in the presence of 10 mM of amino acid, shown in ΔTm, is plotted on the full-length receptor responses to the same concentration of amino acid, shown in ΔRFU. (B) The affinities to the LBD estimated by the DSF (pKd-app = log 1/Kd-app) were plotted on the estimated amino acid potencies for the receptor activation (pEC50-est = log 1/EC50-est, estimated from the responses at 10 mM concentration). (C) The affinities to the LBD estimated by the DSF (pKd-app) were plotted on the estimated amino acid potencies for the receptor activation (pEC50-est, estimated from the responses at 5 mM concentration). (PDF) [file pone.0218909.s003.pdf]
