# Supplementary material for: Differential scanning fluorimetric analysis of the amino-acid binding to taste receptor using a model receptor protein, the ligand-binding domain of fish T1r2a/T1r3
Source: PLoS One. 2019 Oct 4;14(10):e0218909. doi: 10.1371/journal.pone.0218909 (PMC6777825; doi:10.1371/journal.pone.0218909)
Supplement: S1 Table — (PDF) [file pone.0218909.s004.pdf]

S1 Table. ΔT_m and derived $K_{d\text{-app}}$ values estimated from the DSF results of T1r2a/T13LBD at a single ligand concentration.

	ΔT_m (K)†			$K_{d\text{-app}}$ (μM)‡ (fold difference compared with FRET EC_{50})		
Conc.	0.1 mM	1 mM	10 mM	0.1 mM	1 mM	10 mM
Gly	-0.04 ± 0.12	-0.15 ± 0.07	5.08 ± 0.06	–	–	**2220** (0.359)
L-Ala	0.41 ± 0.09	6.92 ± 0.03	11.1 ± 0.02	664 (4.71)	**110** (0.782)	269 (1.91)
L-Ser	-0.14 ± 0.03	-1.20 ± 0.03	7.32 ± 0.06	–	–	**956**
L-Thr	-0.06 ± 0.09	-1.27 ± 0.08	6.99 ± 0.09	–	–	**1080**
L-Asn	0.12 ± 0.07	0.38 ± 0.06	4.36 ± 0.99	2400	7330	**3000**
L-Gln	6.26 ± 0.06	10.80 ± 0.05	13.6 ± 0.02	**14.1** (1.22)	29.2 (2.54)	119 (10.4)
L-Asp	-0.06 ± 0.17	-1.71 ± 0.19	0.74 ± 0.05	–	–	**34900**
L-Glu	0.55 ± 0.26	0.48 ± 0.14	7.06 ± 0.08	488 (0.456)	5700 (5.32)	**1050** (0.982)
L-Lys	-0.04 ± 0.03	-0.40 ± 0.17	-3.83 ± 0.30	–	–	–
L-Arg	0.45 ± 0.17	6.98 ± 0.08	11.0 ± 0.05	608 (3.20)	108 (0.568)	**274** (1.44)
L-Val	0.11 ± 0.24	-0.77 ± 0.14	7.85 ± 0.03	2630	–	**793**
L-Leu	0.08 ± 0.09	-0.94 ± 0.07	6.91 ± 0.05	3490	–	**1110**
L-Ile	-0.50 ± 0.11	-0.57 ± 0.07	2.89 ± 2.40	–	–	**6040**
L-Met	0.45 ± 0.11	6.17 ± 0.05	8.85 ± 0.06	604	146	**562**
L-His	-3.75 ± 0.33	1.21 ± 0.02	5.81 ± 0.07	–	1960	**1670**
L-Phe	0.30 ± 0.04	1.04 ± 0.12	3.53 ± 0.05	918	2370	**4370**
L-Pro	-0.15 ± 0.04	0.04 ± 0.07	7.24 ± 0.02	–	79900	985
D-Ala	0.15 ± 0.05	-0.33 ± 0.07	-1.03 ± 0.09	1950	–	–

†Average ± s.e.m., $n = 4$. ‡Amino acid concentration and average ΔT_m are substituted with Equation 1 described in Materials and Methods. The values written in bold letters are used for further QSAR analysis.
