# Supplementary material for: Differential scanning fluorimetric analysis of the amino-acid binding to taste receptor using a model receptor protein, the ligand-binding domain of fish T1r2a/T1r3
Source: PLoS One. 2019 Oct 4;14(10):e0218909. doi: 10.1371/journal.pone.0218909 (PMC6777825; doi:10.1371/journal.pone.0218909)
Supplement: S2 Table — (PDF) [file pone.0218909.s005.pdf]

S2 Table. ΔRFU and derived $EC_{50\text{-est}}$ values derived from the response assay of T1r2a/T1r3.

	ΔRFU[†]		$EC_{50\text{-est}}$ (mM)		$pEC_{50\text{-est}}$	
Conc.	5 mM	10 mM	5 mM	10 mM	5 mM	10 mM
Gly	13.4 ± 3.45	46.2 ± 9.00	33.8	12.6	1.47	1.90
L-Ala	64.7 ± 12.8	90.8 ± 9.46	3.06	1.49	2.51	2.83
L-Ser	11.5 ± 2.07	33.5 ± 7.59	40.5	21.1	1.39	1.67
L-Thr	3.20 ± 1.51	18.2 ± 6.01	158	47.4	0.801	1.32
L-Asn	0.460 ± 2.58	15.7 ± 6.13	1130	56.3	-0.0524	1.25
L-Gln	104.3 ± 4.24	81.0 ± 4.64	–	2.88	–	2.54
L-Asp	-4.00 ± 1.10	-9.43 ± 1.83	–	–	–	–
L-Glu	3.73 ± 1.63	30.3 ± 1.85	135	24.4	0.871	1.61
L-Lys	8.68 ± 4.29	18.5 ± 8.01	55.1	46.5	1.26	1.33
L-Arg	68.2 ± 5.39	63.4 ± 9.85	2.65	6.44	2.58	2.19
L-Val	3.89 ± 1.23	32.4 ± 7.56	129	22.2	0.890	1.65
L-Leu	0.933 ± 1.91	27.8 ± 5.89	554	27.5	0.257	1.56
L-Ile	2.90 ± 2.82	30.5 ± 6.26	175	24.2	0.757	1.62
L-Met	-2.52 ± 1.72	21.8 ± 7.73	–	37.9	–	1.42
L-His	4.72 ± 2.39	14.4 ± 6.62	105	62.4	0.977	1.20
L-Phe	2.08 ± 2.32	22.4 ± 5.78	246	36.5	0.609	1.44
L-Pro	2.90 ± 1.93	45.1 ± 7.06	175	13.2	0.758	1.88
D-Ala	7.08 ± 2.16	23.6 ± 8.83	68.7	34.2	1.16	1.47

[†]Average ± s.e.m., n = 12 (for L-glutamine at 5 mM), or n = 6 (for others).
