# Supplementary material for: Differential scanning fluorimetric analysis of the amino-acid binding to taste receptor using a model receptor protein, the ligand-binding domain of fish T1r2a/T1r3
Source: PLoS One. 2019 Oct 4;14(10):e0218909. doi: 10.1371/journal.pone.0218909 (PMC6777825; doi:10.1371/journal.pone.0218909)
Supplement: S3 Table — (PDF) [file pone.0218909.s006.pdf]

**S3 Table. Affinities to mfT1r2a/T1r3LBD derived from the DSF results and physicochemical parameters for the α-substituent group of amino acids.**

| | Affinity | Physicochemical parameters | | | | | | |
|---|---|---|---|---|---|---|---|---|
| L-amino acid | $pK_{d\text{-app}}$[a] | $\pi_{\alpha}$[b] | $E_s'^{c}$[c] | HP[d] | $\log K_d$_CW[e] | ΔMgVol[f] | Hydropathy Index[g] | pI[h] |
| Gly | 2.65 | 0 | 0 | 2.39 | -0.69 | 0 | -0.4 | 5.97 |
| Ala | 3.95 | 0.28 | -0.2 | 1.94 | -1.33 | 0.32 | 1.8 | 6 |
| Ser | 3.02 | -0.19 | -0.48 | -5.06 | 2.49 | 0.38 | -0.8 | 5.68 |
| Thr | 2.97 | 0.07 | -0.73 | -4.88 | 1.89 | 0.52 | -0.7 | 5.6 |
| Asn | 2.52 | -0.52 | -0.98 | -9.68 | 4.88 | 0.58 | -3.5 | 5.41 |
| Gln | 4.85 | -0.52 | -0.82 | -9.38 | 4.07 | 0.72 | -3.5 | 5.65 |
| Asp | 1.46 | 0.05 | -0.98 | -10.95 | 3.27 | 0.53 | -3.5 | 2.77 |
| Glu | 2.98 | 0.02 | -0.82 | -10.2 | 2.25 | 0.68 | -3.5 | 3.22 |
| Arg | 3.56 | -0.42 | -0.82 | -19.92 | 4.32 | 1 | -4.5 | 10.76 |
| Val | 3.10 | 1 | -1.29 | 1.99 | -2.97 | 0.6 | 4.2 | 5.96 |
| Leu | 2.96 | 1.52 | -1.44 | 2.28 | -3.62 | 0.74 | 3.8 | 5.98 |
| Ile | 2.22 | 1.43 | -1.81 | 2.15 | -3.62 | 0.74 | 4.5 | 6.02 |
| Met | 3.25 | 0.99 | -1.03 | -1.48 | -1.73 | 0.76 | 1.9 | 5.74 |
| His | 2.78 | 0.17 | -0.86 | -10.27 | 3.34 | 0.75 | -3.2 | 7.59 |
| Phe | 2.36 | 1.78 | -0.9 | -0.76 | -2.19 | 0.93 | 2.8 | 5.48 |

[a]Converted from the values in Supplementary Table 1. [b]For hydrophobicity. Cited from Akamatsu *et al., J. Pharm. Sci.*, 83, 1026 (1994). [c]For steric effects. Cited from Akamatsu *et al., J. Pharm. Sci.*, 83, 1026 (1994). [d]For hydration potential. Cited from Wolfenden *et al., Biochemistry,* 20, 849 (1981). [e]For polarity. Cited from Radzicka & Wolfenden, *Biochemistry*, 27, 1664 (1988), free energies for transfer from cyclohexane to an aqueous solution. [f]McGowan volumes [ref.: McGowan, *J. Appl. Chem. Biotechnol.*, 28, 599 (1978)] calculated by BioLoom (BioByte, Claremont, CA, USA). [g]For hydrophobicity/hydrophilicity. Cited from Kyte & Doolittle, *J. Mol. Biol.*, 157, 105 (1982). [h]Cited from Handbook of Chemistry and Physics, CRC Press.
